# Periprosthetic joint infection as the leading indication associated with in-hospital death after revision hip and knee arthroplasty: a ten-year single-centre retrospective cohort study of 103 in-hospital deaths among 2495 patients

**DOI:** 10.1186/s13018-026-07259-4

**Published:** 2026-10-01

**Authors:** Annemarie Rusche, Andreas Roth, Christina Pempe

**Affiliations:** Department of Orthopaedics, Trauma and Plastic Surgery, University Hospital Leipzig, Liebigstrasse 20, 04103 Leipzig, Germany

**Keywords:** Arthroplasty, replacement, hip, Arthroplasty, replacement, knee, Reoperation, Prosthesis-related infections, Hospital mortality

## Abstract

**Objective:**

Revision arthroplasty procedures are associated with increased in-hospital morbidity and mortality. This study aimed to characterise in-hospital death after revision hip and knee arthroplasty, with particular focus on periprosthetic joint infection (PJI) as the leading indication among decedents.

**Methods:**

In this single-centre retrospective cohort study, all patients who died in hospital after revision hip or knee arthroplasty between 2014 and 2024 were identified among 2495 patients with revision surgery coded as OPS 5-821* and 5-823*. Primary procedures and patients younger than 18 years were excluded. Clinical and microbiological data of these 103 decedents were reviewed to characterise the circumstances, timing and causes of in-hospital death, including ICU admission, septic shock, and reoperation. Clinical characteristics, causes of death, and treatment strategies were described according to revision indication for periprosthetic joint infection (PJI), periprosthetic fracture (PF), and other indications.

**Results:**

Of 2495 patients undergoing revision surgery over the study period, 103 patients (4.1%) died in hospital. Decedents were predominantly elderly, with a median age of 80 years (IQR 70–85) and a higher proportion of women (58.3%) than men (41.7%). PJI was the indication for revision in the majority of decedents (*n* = 78/103, 75.7%), and sepsis was the leading cause of death overall (*n* = 69/103, 67.0%) and among patients with PJI (*n* = 64/78, 82.1%). Patients with PJI had elevated CRP-level, with median value of 260.0 mg/l (IQR 148.5-339.3) and nearly half showed polymicrobial joint infection (*n* = 36/78, 46.2%).

**Conclusion:**

PJI was the leading indication among patients who died in hospital after revision arthroplasty. Positive blood cultures, elevated preoperative CRP, and polymicrobial infection characterised this cohort of fatal cases and may reflect greater disease severity, although this descriptive analysis cannot establish them as independent risk factors. Comparative studies including surviving patients are needed to confirm which characteristics independently predict adverse outcomes and to inform perioperative risk stratification and surgical decision-making.

## Introduction

Revision surgery after hip or knee arthroplasty is increasingly common due to the growing number of primary arthroplasties and aging population [1]. Although advances in surgical techniques, perioperative care, and infection management have improved outcomes, revision procedures remain associated with substantial morbidity and mortality [2, 3]. Among several causes for revision, periprosthetic joint infection (PJI) has been reported to increase perioperative risk [4, 5]. Previous studies have demonstrated that one-year mortality after revision arthroplasty can reach up to 14%, with PJI accounting for large proportion of these cases [6–8]. Revision arthroplasty imposes a substantial and growing economic and clinical burden, with recent national registry analyses confirming markedly higher complication rates, costs, and length of stay compared with primary arthroplasty [9, 10]. Established risk factors for revision and its complications include e.g. diabetes [11] and obesity [12]. Most of these factors have been derived from registry data lacking perioperative and especially microbiological information. The present study was therefore designed to provide a comprehensive, single-centre analysis of in-hospital deaths following hip and knee arthroplasty revision surgery over a ten-year period in a high-volume orthopaedic hospital. The aims were (i) to analyse the indications for revision among decedents, (ii) to characterise patient demographics and comorbidities, (iii) to describe the timing of in-hospital deaths and their main causes, and (iv) to describe the clinical and microbiological characteristics associated with fatal outcomes. A better understanding of these factors could help clinicians to recognise elevated risk earlier and adjust surgical and antimicrobial management accordingly.

## Materials and methods

### Study design and patient selection

**Fig. 1:**
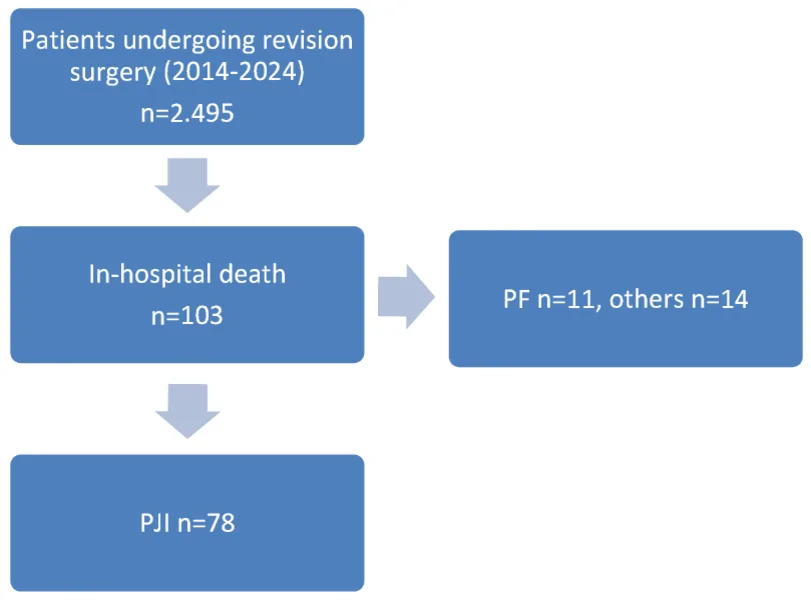
Patient selection. 2495 Patients undergoing revision surgery between 2014 and 2024 were identified. 103 patients, who died during hospitalization were further assessed. Patients revised for periprosthetic fracture or other reasons were analysed separately. PJI was defined using MSIS criteria (≥ 1 major criterion or ≥ 6 points), these patients (*n* = 78) underwent further analysis. Others = Hip arthroplasty Dislocation, Aseptic Loosening, Postoperative Haematoma, Traumatic Wound Dehiscence, Suspected PJI. PF=Periprosthetic Fracture, PJI=Periprosthetic Joint Infection, MSIS=Musculoskeletal Infection Society

This single-center retrospective cohort study included all patients who underwent revision surgery for hip or knee arthroplasty between 2014 and 2024 and died during hospital stay. Patients undergoing primary hip or knee arthroplasty were excluded from the analysis. Patients were identified retrospectively from the hospital’s electronic archive based on procedure coding. During the study period (2014–2024), all patients coded for hip or knee arthroplasty revision of any kind were screened for in-hospital death. A total of 2,495 patients underwent revision surgery for hip or knee arthroplasty during the study period (Fig. 1). Patients requiring revision surgery during the same hospitalization following primary implantation were also included. Each patient was analysed only once in this study, even when more than one revision was performed during the study period. Patients undergoing revision surgery for knee and hip arthroplasty were counted only once. Classification was based on the procedure requiring the more extensive surgical intervention.

### Data acquisition

Patients were identified using OPS codes 5-821* and 5-823*. For simplification, the term “hip arthroplasty” refers to all total hip replacements, including hemiarthroplasty, while “knee arthroplasty” contains all knee replacements and knee arthrodesis procedures. There were no patients with unicompartmental knee replacements in this cohort. Of 103 patients, 28 underwent revision of knee arthroplasty and 75 of hip arthroplasty. Out of these 75 patients, one patient underwent revision of both knee and hip arthroplasty and was counted among hip arthroplasty because it required more extensive surgical treatment. Of the 103 decedents, hemiarthroplasty accounted for 16 of the hip arthroplasty group and arthrodesis accounted for 2 of the knee arthroplasty group. All data were extracted from existing patient records, including patient demographics (age, sex), body mass index (BMI), Charlson Comorbidity Index (CCI), American Society of Anesthesiologists (ASA) classification, indication for revision, surgical treatment strategy, microbiological findings, preoperative CRP, ICU admission, septic shock, reoperation, and the timing and cause of death. Timing of death was defined as time between last orthopaedic revision surgery and in-hospital death. Sepsis and cause of death were classified according to the documentation in the medical record and discharge letter, as well as laboratory and microbiological findings, without retrospective reclassification using standardised criteria. Microbiological findings were separated in two subgroups: blood cultures and joint infections, found intraoperatively or via joint aspiration. PJI diagnosis was assessed using Musculoskeletal Infection Society (MSIS) guidelines [13]. Patients with at least one major criterion or patients scoring at least 6 points were defined as PJI. MSIS criteria were chosen as the diagnostic standard, as they were the internationally validated criteria in routine use throughout the study period.

### Statistical analysis

Data were managed using Excel database (Microsoft Corp., Redmond, WA, USA) and exported to SPSS 29.0 (IBM Corp., Armonk, NY, USA). Data were assessed for normal distribution using Shapiro-Wilk-Test. Continuous variables with normal distribution were presented as means ± standard deviation (SD), otherwise they were presented as median and interquartile range IQR 25th -75th percentile. Categorical variables were reported as absolute numbers (n) and relative numbers (%).

## Results

### Demographics

Overall, 103 patients (4.1%) died postoperatively during hospital stay following revision surgery. The median age of this decedent cohort was 80 years (IQR 70–85) with women more frequently affected than men (58.3% vs. 41.7%). Median BMI was 28.4 kg/m² (IQR 24.8–32.5), median CCI was 6 (IQR 4–7), and median ASA classification was 3 (IQR 3–3). These parameters are reported descriptively, no statistical testing for independence or between-group differences was performed. Numerically, ASA classification appeared similar between patients undergoing revision for hip versus knee arthroplasty and across revision indications. Patients undergoing revision for hip arthroplasty had a median BMI of 27.3 kg/m² (IQR 24.2–31.2), compared with 32.0 kg/m² (IQR 27.7–38.8) in those undergoing revision for knee arthroplasty. Median CCI was 6 (IQR 4–7) in patients with hip arthroplasty and 4.5 (IQR 4–6) in patients with knee arthroplasty. Patients undergoing revision for PJI had a median CCI of 5 (IQR 4–7), compared with 7 (IQR 5–7) in patients with PF. Median BMI was 30.0 kg/m² (IQR 26.1–34.1) in patients undergoing revision for PJI and 26.2 kg/m² (IQR 22.3–28.6) in those with PF.

### Indication for revision surgery and treatment strategies

PJI and PF were the most common indication for revision. Additional revision indications are summarized in Table 1. Explantation of the prosthesis was the most frequent surgical strategy (*n* = 46), particularly among patients with PJI (*n* = 42/78). Revision strategies according to the underlying indication are presented in Table 2.

**Table 1:** Indication for revision surgery

	All patients
Total	103 (100%)
PJI	78 (75.7%)
PF	11 (10.7%)
Dislocation	6 (5.8%)
Aseptic Loosening	3 (2.9%)
Postoperative Haematoma	2 (1.9%)
Traumatic Wound Dehiscence	1 (1.0%)
Suspected PJI	2 (1.9%)

PJI=Periprosthetic Joint Infection, PF=Periprosthetic Fracture, MSIS=Musculoskeletal Infection Society

Two patients classified as suspected PJI did not meet MSIS diagnostic criteria and are therefore grouped under ‘other causes’ in Table 2. Data are reported as absolute numbers (n) and relative numbers (%)

**Table 2:** Treatment strategies

	All patients *n* = 103	PJI *n* = 78	PF *n* = 11	other causes *n* = 14
Explantation	46 (44.7%)	42 (53.8%)	–	4 (28.6%)
of which: explantation following an initial failed DAIR retention attempt	–	22 (52.4%)	–	–
DAIR	28 (27.2%)	27 (34.6%)	–	1 (7.1%)
Prosthesis replacement	18 (17.5%)	–	10 (90.9%)	8 (57.1%)
ORIF	1 (1.0%)	–	1 (9.1%)	–
Other treatment	10 (9.7%)	9 (11.5%)	–	1 (7.1%)

The reported percentages for these subcategories refer to the total number of patients within each revision category, not to the overall patient population. Data are reported as absolute numbers (n) and relative numbers (%)

DAIR=debridement, antibiotics and implant retention. ORIF=open reduction and internal fixation using a plate, PJI=Periprosthetic Joint Infection, PF=Periprosthetic Fracture

Revision was performed due to PJI, PF and other causes, including dislocation, aseptic loosening, hematoma and traumatic wound dehiscence. Other treatments comprise incomplete explantation, reimplantation and construction of a stable fistula. Other causes are further described in Table 1. The sub-row “of which: explantation following an initial failed DAIR retention attempt” indicates, for the Explantation row, how many patients were initially managed with a DAIR retention attempt that subsequently failed, requiring explantation in a second revision; the denominator for this sub-row percentage is the number of explantations in that column.

### Hospital stay

Median length of hospital stay was 21 days (IQR 12–42). Median interval between last orthopaedic revision surgery and death was 9 days (IQR 2–17). The majority of deaths (*n* = 34, 33%) occurred within the first three postoperative days. Sixteen patients (15.5%) died between postoperative day four and seven, 24 patients (23.3%) within the first two weeks and 29 patients (28.1%) died more than two weeks after orthopaedic revision surgery.

### Cause of death

The majority of patients (*n* = 69, 67.0%) died due to sepsis with subsequent multi-organ failure. Other causes of death are summarized in Table 3.

**Table 3:** Perioperative causes of death

	All patients *n* = 103	PJI *n* = 78	PF *n* = 11	Other causes *n* = 14
Sepsis	69 (67.0%)	64 (82.1%)	2 (18.1%)	3 (21.4%)
Cardiopulmonary decompensation	15 (14.6%)	5 (6.4%)	5 (45.5%)	5 (35.7%)
Respiratory failure	7 (6.8%)	2 (2.5%)	2 (18.2%)	3 (21.4%)
Pulmonary embolism	3 (2.9%)	2 (2.5%)	–	1 (7.1%)
Haemorrhagic shock	3 (2.9%)	1 (1.2%)	1 (9.1%)	1 (7.1%)
Myocardial infarction	2 (1.9%)	1 (1.2%)	–	1 (7.1%)
Pneumonia	2 (1.9%)	1 (1.2%)	1 (9.1%)	–
Carcinoma	1 (1.0%)	1 (1.2%)	–	–
Progression of neurodegenerative disease	1 (1.0%)	1 (1.2%)	–	–

PF=Periprosthetic Fracture, PJI=Periprosthetic Joint Infection

Data is presented in absolute (n) and relative numbers (%)

Patients were further categorized for cause of revision (PJI, PF and other causes). The patient categorised under “progression of neurodegenerative disease” presented after a fall and ultimately died with persistent impairment of consciousness in the context of this underlying condition. The patient with carcinoma had known metastatic breast cancer and was managed under a palliative treatment concept.

### Periprosthetic joint infection

Majority of patients with PJI (*n* = 64, 82.1%) died due to sepsis and only 14 patients (17.9%) died due to other reasons. Almost all patients (77/78, 98.7%) with PJI required ICU treatment during hospital stay. Preoperative C-reactive protein (CRP) levels were assessed in 76 patients, with a median of 260.0 mg/l (IQR 148.5-339.3). In 5 patients, no blood cultures were obtained, therefore the data was restricted to the remaining 73 patients. Most patients (50/73, 68.5%) had a bloodstream infection. Blood cultures were sterile in 23 patients (23/73, 31.5%). In 73 patients (93.6%) bacteria were found intraoperatively or via joint aspiration. In 4 patients (5.1%) no bacteria were detected during hospital stay. One patient (1.3%) exhibited multiple intraoperative microbiological results with unknown origin.

**Fig. 2:**
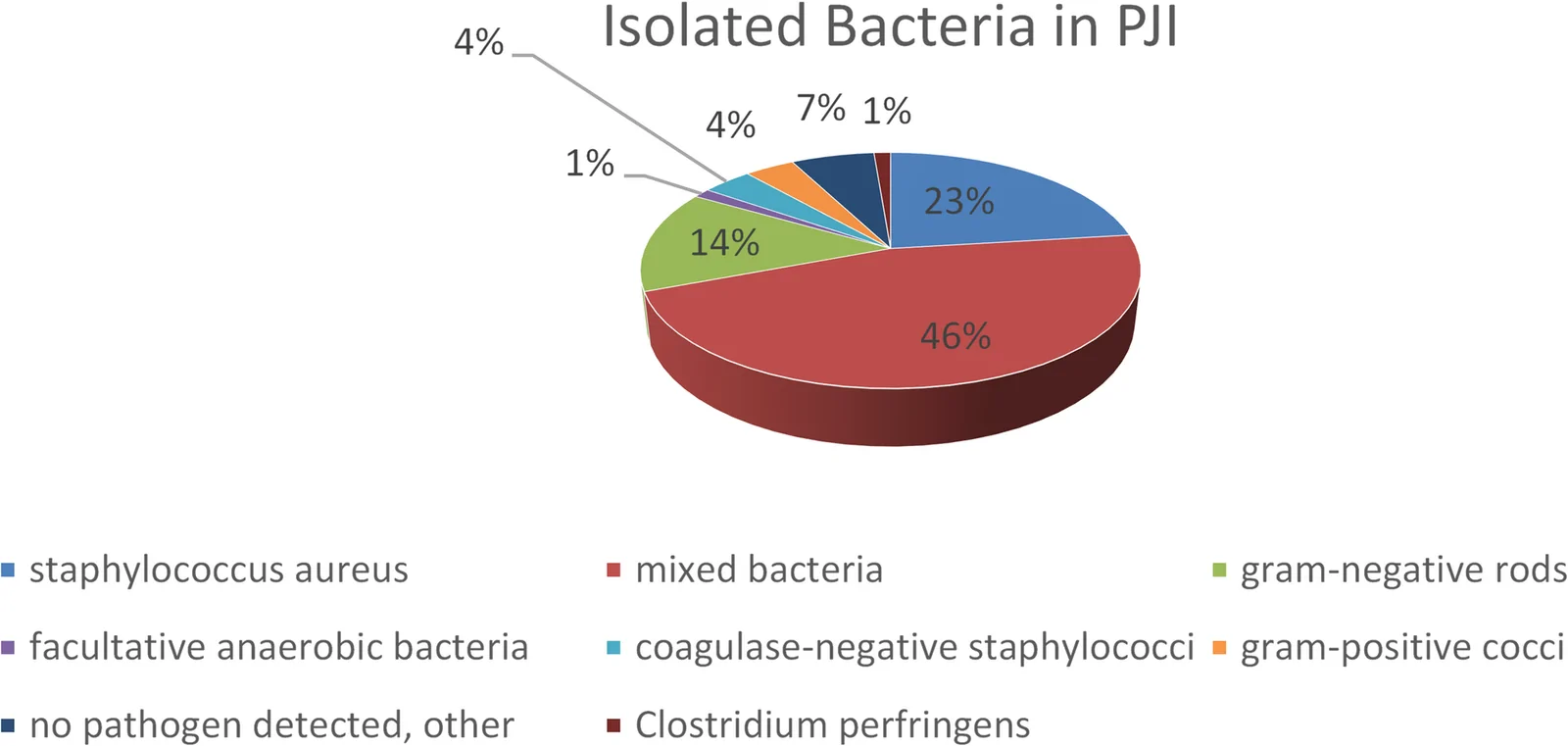
Distribution of bacteria isolated in PJI. Bacteria were found in the affected joint. In 4 patients, no pathogens were detected during hospital stay in our institution. One patient showed pathogen of unknown origin intraoperatively, since multiple surgeries were performed at a time. Bacteria were summarized into subgroups. Owing to the large number of distinct organism combinations observed in polymicrobial infections, these could not be further broken down into individual pathogen combinations; polymicrobial cases are therefore presented as a single “mixed bacteria” category. PJI = Periprosthetic Joint Infection

Nearly half of the patients showed polymicrobial infections (36/78, 46.2%) (Fig. 2).

## Discussion

This study aimed to investigate perioperative mortality and to further characterize patients undergoing revision surgery for hip and knee arthroplasty. Patients who died postoperatively after revision surgery were predominantly elderly. Most patients underwent revision for PJI (*n* = 78/103) and main cause of death was associated with sepsis (*n* = 64/78). Patients with PJI presented elevated preoperative CRP levels with a median of 260.0 mg/l (IQR 148.5-339.3) and polymicrobiological infection of the joint (*n* = 36/78).

In this study, the present cohort included predominantly elderly patients which were older compared to populations reported in the current literature [2, 4, 14]. Advanced age, particularly above 80 years, has been consistently identified as a significant risk factor for higher perioperative mortality [2, 3, 15].

Most patients in this cohort underwent revision surgery due to PJI and subsequently died as a result of septic shock with multiorgan failure. Consistent with this observation, sepsis and organ failure have been identified as significant contributors to an increased in-hospital mortality [15] and revision for PJI itself is an independent risk factor for higher perioperative mortality [3, 16, 17].

However, the available literature regarding the exact causes of death following revision for PJI remains limited and heterogeneous. While septic shock has been described as the most common cause of death in some studies [14, 18], others report that malignancies, as well as circulatory and respiratory diseases, myocardial infarction, pulmonary embolism or pneumonia account for the majority of deaths [6]. These differences may be explained by variate study designs and data sources. Registry-based studies may lack precise information on the cause of death compared to single-center analyses. Additionally, variability in the definition of PJI and differences in disease severity across studies further complicate direct comparisons. In this study, most patients needed ICU treatment during hospital stay. This finding is consistent with the results reported by Prange et al. [18], whereas Reinhard et al. described markedly lower ICU admission rates of approximately 25% [15]. Such differences are likely attributable to variations in the severity of infection and the corresponding need for more extensive surgical and medical management.

In the present cohort of decedents, most patients with PJI also had bloodstream infections. The reported prevalence of bloodstream infection in the literature varies widely, ranging from 17% to 66.7% [14, 19]. Kuo et al. identified positive bloodstream cultures as an independent risk factor for treatment failure following DAIR [20]. However, positive blood cultures may also indicate acute hematogenous infections, for which better outcomes with DAIR have been reported in literature when comparing late chronic infection [21]. To distinguish between these two scenarios would require reliable information on symptom onset. As in most retrospective studies, this information could not be established in the present cohort. The presence of bloodstream infection should therefore be interpreted in the overall clinical context when considering treatment strategy.

Preoperative C-reactive protein (CRP) levels were markedly elevated compared to values reported in the literature, which typically range from 62 to 131 mg/L [19, 22], which may be an indicator for severity of infection in this cohort. Baertl et al. ^19^ demonstrated that patients classified as septic exhibited higher CRP levels, and Ludwick et al. ^23^ identified elevated CRP as an independent risk factor for systemic sepsis and persistent PJI. The markedly elevated CRP levels observed in this cohort of decedents may reflect greater disease severity; however, no validated cut-off value linking CRP to a specific surgical strategy has been established in the literature, and this descriptive, decedent-only cohort cannot determine whether CRP should inform treatment decisions. Microbiological findings in this study showed a predominance of polymicrobial infections based on joint aspiration and intraoperative cultures. This contrasts with existing literature, where monomicrobial infections, most commonly involving *Staphylococcus aureus* and coagulase-negative staphylococci, are typically reported [14, 19, 23]. In literature, the prevalence of polymicrobial infections has been described to range between 16% and 32%. While Baertl et al.^19^ did not observe an increased rate of polymicrobial infections among septic patients, Ludwick et al. [23] reported an association between polymicrobial infections and higher rates of persistent infection. Additionally, polymicrobiological infection have higher risk for DAIR treatment failure [24]. Polymicrobial infections are generally considered more difficult to treat, often requiring broader and prolonged antibiotic regimens and repeated surgical interventions. Moreover, they may be associated with an increased risk of systemic infection and septic multiorgan failure.

Beyond the microbiological and inflammatory characteristics described above, several patient-level factors that are established contributors to revision arthroplasty and its complications in the wider literature, e.g. obesity [12], could only be assessed descriptively in the present cohort. No comparator cohort of survivors was available and no formal testing for subgroup differences or confounding was performed. Descriptively, patients revised for knee arthroplasty had a numerically higher median BMI than those revised for hip arthroplasty (32.0 vs. 27.3 kg/m²), and patients revised for PJI had a numerically higher median BMI than those revised for PF (30.0 vs. 26.2 kg/m²), consistent with the association between obesity and PJI.

Given that obesity has been associated with both a higher risk of PJI and more frequent polymicrobial infection [12], unmeasured confounding by these factors cannot be excluded and should be addressed in future comparative studies. Higher median CCI in patients undergoing revision for hip arthroplasty may be partly explained by the inclusion of patients with hemiarthroplasty, who typically have more comorbidities compared to those with total hip replacement, which is why this surgical strategy was chosen in the first place [25]. Furthermore, the hip arthroplasty group therefore also comprises patients undergoing revision after femoral neck fracture rather than primary elective arthroplasty alone.

Nevertheless, several limitations of this study must be acknowledged. First, and most importantly, this analysis included only the 103 patients who died in hospital, without a comparator group of surviving revision patients; consequently, the clinical and microbiological characteristics described here, including hematogenous infection, elevated CRP, and polymicrobial infection, should be interpreted as characteristics of fatal cases rather than as independently validated risk factors for mortality, and the implications for treatment strategy discussed above cannot be generalised beyond this descriptive cohort. Second, only in-hospital deaths were assessed. Patients who died after discharge were not included, which may underestimate overall perioperative mortality and bias the observed causes and timing of death toward early, in-hospital events. Third, this analysis relied on identification by procedure coding, which is susceptible to miscoding or incomplete capture, and hip and knee revisions, as well as hemiarthroplasty and arthrodesis, were analysed together rather than stratified by joint or subgroup, although these differ in microbiology, prognosis and revision strategy. Fourth, comorbidity data (BMI, CCI and ASA classification) were available and are reported descriptively in the Results. However, no comparator cohort of survivors was available and no formal statistical testing for between-group differences or confounding was performed, so these descriptive comparisons cannot establish whether comorbidity burden independently distinguishes sepsis-related deaths from decompensation of pre-existing disease. Fifth, the time from index arthroplasty to onset of infection was not available, precluding classification of PJI as acute or chronic, which is a key determinant of DAIR appropriateness and may confound interpretation of the proposed severity indicators. As such, the findings may not be generalizable to the broader population of patients undergoing revision hip and knee arthroplasty. Sixth, the retrospective study design is associated with limitations in data completeness and accuracy. Missing information in selected variables constrains the extent of subsequent analyses, while the lack of a control cohort prevents the establishment of comparative or causal relationships. Moreover, key clinical parameters, e.g. the classification of patients as septic, were derived exclusively from existing documentation, precluding standardized clinical reassessment. Furthermore, comprehensive analysis of all patients undergoing revision surgery, including regression analysis adjusted for age, comorbidity and indication, would be necessary to establish independent risk factors for perioperative mortality. The present study design does not allow for such conclusions but rather provides descriptive insights and observations regarding treatment strategy. Finally, this study was conducted at a single tertiary referral centre. The notably high proportion of polymicrobial infection and ICU admission observed here may partly reflect referral bias, case complexity, or institutional admission thresholds rather than a true epidemiological difference from other populations, and this should be considered when comparing these findings with other cohorts. 

This manuscript adheres to the STROBE (Strengthening the Reporting of Observational Studies in Epidemiology) guidelines for reporting observational cohort studies.

## Conclusion

In this study, PJI was the leading indication among patients who died in hospital following revision arthroplasty. Positive blood cultures, elevated preoperative CRP levels and polymicrobial infection were common in this group. This may reflect greater disease severity, though this descriptive analysis cannot establish them as independent risk factors. These characteristics were particularly common among elderly patients with PJI in this cohort. Comparative studies including surviving patients are needed to confirm these associations and to identify independent risk factors for mortality after revision surgery.

## Data Availability

The datasets analysed during the current study are not publicly available due to patient privacy and institutional data protection regulations but are available from the corresponding author on reasonable request, subject to approval by the Institutional Ethics Committee and the respective institution.
